# East Line Medical Association

**Published:** 1885-12

**Authors:** J. W. Garnett

**Affiliations:** Secretary


### Society Notes.

### EAST LINE MEDICAL ASSOCIATION (TEXAS).

This Association is composed of several counties in East Texas, and is in a flourishing condition. It has a membership of forty-seven, and is doing some good work—an example we would be pleased to see followed.

At a meeting held at Greenville, Oct. 6, the following by Dr. J. J. Dial was unanimously adopted and ordered published in Daniel's Texas Medical Journal

*Whereas*, The American Medical Association at its meeting held in the City of Washington, in May, 1884, appointed a committee to invite the "International Medical Congress" to hold its next meeting at the City of Washington, said committee having other powers delegated to them; and, whereas, the American Medical Association, at its meeting held in the City of New Orleans, in April last did, as it had a right to do, receive such portions of said committee's work as was satisfactory to it, and enlarged said committee, making it, what it should be, national in its character. And, whereas, certain members of the profession have objected thereto and have done what they could to throw obstacles in the way of a perfect and harmonious organization of the present committee and the work of said committee; and, whereas, we believe it to be our duty to express our views in the present condition of affairs; therefore be it

*Resolved*, That we do most heartily endorse the action of the American Medical Association, at its meeting at New Orleans, in May last; and also, do most heartily endorse the present committee and its work up to date.

2nd. *Resolved*, That we do hereby pledge ourselves to sustain the American Medical Association in its efforts to make the "International Medical Congress" a success, and to elevate the standard of legitimate medicine in these United States.

5rd. *Resolved*, That we are proud of and do most cordially endorse the delegates from our State Medical Association to the American Medical Association at New Orleans, in May last; we believe their action represents the true sentiments of a very large majority ot the profession in Texas.

4th. *Resolved*, That we disclaim any sectional feeling in this matter. We know no North, no South, no East, no West. We are for honorable medicine — THE AMERICAN MEDICAL ASSOCIATION one and indivisible, now and forever!

5th. *Resolved*, That a copy of these Resolutions be sent to DANIEL'S MEDICAL JOURNAL for publication.

A true copy. J. W. GARNETT, Secretary.

